# Effect of depressive symptom and depressive disorder on glaucoma incidence in elderly

**DOI:** 10.1038/s41598-021-85380-6

**Published:** 2021-03-15

**Authors:** Younhea Jung, Kyungdo Han, Sheng-min Wang, Hye yeon Yoon, Jung Il Moon

**Affiliations:** 1 Department of Ophthalmology, Yeouido St. Mary’s Hospital, College of Medicine, The Catholic University of Korea, 10, 63-ro, Yeongdeungpo-gu, Seoul, 07345 Republic of Korea; 2 Department of Statistics and Actuarial Science, Soongsil University, Seoul, Republic of Korea; 3 Department of Psychiatry, Yeouido St. Mary’s Hospital, College of Medicine, The Catholic University of Korea, Seoul, Republic of Korea

**Keywords:** Epidemiology, Ocular hypertension, Depression

## Abstract

Although depression and glaucoma share several common pathophysiology, the risk of glaucoma in patients with depression has not been reported. Thus, we investigated the effect of depressive symptom and depressive disorder on glaucoma incidence. In this nationwide population-based cohort study, all subjects receiving the National Screening Program at the age of 66 during 2009–2014 were included. These subjects were divided into depression group and no depression group based on subjective depressive symptoms and clinically diagnosed depressive disorder and were tracked until 2017 for development of glaucoma. Of the 922,769 subjects included in the study, 191,636 (20.77%) subjects were categorized as depression group. Subjects with depression showed increased hazard of developing glaucoma (adjusted HR = 1.12[95% confidence interval (CI), 1.09–1.15]) than those without depression. The risk of glaucoma increased sequentially from those with no depression to those with subjective depressive symptom (adjusted HR = 1.09[95% CI, 1.06–1.13]), those with clinically diagnosed depressive disorder (adjusted HR = 1.23[95% CI, 1.14–1.32]), and those with both subjective depressive symptom and clinically diagnosed depressive disorder (adjusted HR = 1.36[95% CI, 1.22–1.52]). Our analyses suggest that individuals with depression had a greater risk of developing glaucoma than those without depression. Subjective depressive symptoms and clinically diagnosed depressive disorder independently and synergistically increased the risk of glaucoma incidence.

## Introduction

Depression is a highly prevalent disease, projected to be one of the three causes of burden of disease in 2030 worldwide^1^. Depression has been recognized as an important risk for other chronic diseases including coronary artery disease^2–4^, hypertension^5–7^, diabetes^8–10^, and migraine^2,11^ through the autonomic nervous system and inflammation, which are also associated with glaucoma^12,13^. Glaucoma is the leading cause of irreversible blindness worldwide, characterized by progressive death of retinal ganglion cells (RGC)^14^.

There are prior studies reporting strong association between depression and glaucoma^15–20^. Zhang et al.^15^ have reported that the odds ratio for having depression and also glaucoma was very high (12.3). Wang et al.^18^ reported that prevalence of depression was higher in those with glaucoma (10.9%) than those without (6.9%). However, the majority of the studies report only cross-sectional association between depression and glaucoma^18–21^, and others have focused on the risk of depression among patients with glaucoma^17,19,21^. Little research is available on the risk of glaucoma in patients with depression. Jampel et al.^22^ previously reported that depression was related with worse visual function in glaucoma patients, but to our knowledge, no study has explored the risk of glaucoma in patients with depression.

Several hypotheses may account for the risk of glaucoma in patients with depression. First, disrupted neuroplasticity in depression with diminished level of essential trophic factors such as brain-derived neurotrophic factor (BDNF) may contribute to the death of retinal ganglion cells (RGC) in glaucoma^23–26^. Previous studies have identified loss of synapses and changes in connectivity of neural networks in depressive disorder^27,28^. This may contribute to the death of retinal ganglion cells (RGC) in glaucoma as deprivation of retrograde axonal transport contributes to RGC loss in glaucoma^26^. Second, neuroinflammation and microglial activity have been found in the brains of patients with depression^29^, and there is mounting evidence that neuroinflammation plays a role in the pathogenesis of glaucoma^30–32^. These shared common biological pathways could account for the risk of glaucoma in depression patients. Furthermore, autonomic nervous system function is often altered in depression, which seems to be the link between depression and other physical dysfunctions^33–35^. Autonomic dysfunction has also been associated with glaucoma, especially those with normal tension, which is the most prevalent type of glaucoma in Korea^36,37^. This may also partly account for the increased risk of glaucoma in depression patients.

Therefore, in this nationwide population-based cohort study, we explored the risk of patients developing glaucoma after being diagnosed with depression using the Korean National Health Information Database (KNHID) to explore whether depression, either subjective or objective, increased the risk of glaucoma incidence.

## Results

A total of 922,769 subjects were included in the study: 731,133 (79.23%) subjects in the no depression group, indicating no clinically diagnosed depressive disorder and no subjective depressive symptom at the time of National Screening Program for Transitional Ages (NSPTA) and 191,636 (20.77%) subjects in the depression group, indicating either a history of clinically diagnosed depressive disorder or subjective depressive symptom. The demographic characteristics and clinical data of patients with and without depression are shown in Table 1. The depression group had more subjects who were females, from lower income group, and had diabetes, hypertension, and hyperlipidemia. In total, 27,286 (2.96%) subjects had glaucoma (Supplementary Table 1).

**Table 1:** Demographic and clinical data of study subjects who underwent National Screening Program for Transitional Ages at age 66.

n (%)	Depression					
	No 731,133	Yes 191,636	p	Cohen *d*		
Age	66 ± 0	66 ± 0				
Gender, Male	375,964 (51.42)	79,399 (41.43)	< .0001	0.0811		
Income (low)	209,941 (28.71)	56,079 (29.26)	< .0001	0.0049		
Diabetes (yes)	139,174 (19.05)	40,885 (21.35)	< .0001	0.0236		
Hypertension (yes)	387,050 (52.99)	104,419 (54.54)	< .0001	0.0126		
Hyperlipidemia (yes)	249,127 (34.1)	72,834 (38.04)	< .0001	0.0335		
Weight (kg)	62.04 ± 9.57	60.91 ± 9.58	< .0001	0.1178		
Height (cm)	159.68 ± 8.37	158.27 ± 8.27	< .0001	0.1696		
Body mass index (kg/m^2^)	24.29 ± 2.98	24.29 ± 3.15	0.8674	0.0004		
Waist circumference (cm)	83.22 ± 8.13	83.1 ± 8.43	< .0001	0.0137		
Triglyceride (mg/dL)	119.95 (119.81–120.09)	121.76 (121.49–122.04)	< .0001	0.0291		
Smoking			< .0001	0.026		
Never	491,087 (67.24)	132,220 (69.14)				
Ex-smoker	137,584 (18.84)	31,294 (16.36)				
Current smoker	101,661 (13.92)	27,725 (14.5)				
Alcohol			< .0001	0.0407		
Non	499,637 (68.67)	139,437 (73.18)				
Mild to Moderate	195,470 (26.86)	43,043 (22.59)				
Heavy	32,498 (4.47)	8,054 (4.23)				
Exercise						
No—Heavy (< 3 days)	580,708 (79.43)	160,390 (83.7)				
Heavy (≥ 3 days) *	150,425 (20.6)	31,246 (16.34)	< .0001	0.0434		
No—Moderate (< 5 days)	538,851 (73.7)	150,635 (78.6)				
Moderate (≥ 5 days) ^†^	192,282 (26.36)	41,001 (21.48)	< .0001	0.0455		
No—Light (< 5 days)	337,353 (46.14)	96,354 (50.28)				
Light (≥ 5 days) ^‡^	393,780 (53.97)	95,282 (49.87)	< .0001	0.0333		

*Heavy exercise indicates exercise that causes one to sweat profusely and produce shortness of breath for at least 20 min a day and more than 3 days a week.

^†^Moderate exercise indicates exercise that causes one to break out in light to moderate sweat or produce mild shortness of breath for at least 30 min a day and more than 5 days a week.

^‡^Light exercise indicates exercise that does not cause sweat or shortness of breath at least 30 min a day and more than 5 days a week.

Table 2 shows the association between depression and glaucoma incidence. Overall, subjects with depression showed increased hazard of developing glaucoma after adjusting for confounding factors including age, gender, smoking, alcohol, physical exercise, income, BMI, hypertension, hypercholesterolemia, and diabetes (adjusted HR = 1.12 [95% confidence interval (CI), 1.09–1.15]) than those without depression. Subjects with depression had increased HR compared to those without regardless of smoking or drinking status (Supplementary Table 2).

**Table 2:** Risk of glaucoma according to subjective depressive symptom or clinically diagnosed depressive disorder.

	N	Glaucoma N (%)	Duration (person -years)	IR per 1000	HR (95% C.I)	
					Model 1	Model 2
No depression group	731,133	20,910 (2.86)	3,791,759.22	5.51459	1 (ref.)	1 (ref.)
Depression group	191,636	6,376 (3.33)	1,014,503.39	6.28485	1.129 (1.098,1.162)	1.120 (1.088,1.152)
Subjective depressive symptoms						
None	752,713	21,659 (2.88)	3,901,038.82	5.55211	1 (ref.)	1 (ref.)
At least one symptom	170,056	5,627 (3.31)	905,223.79	6.21614	1.108 (1.075,1.141)	1.100 (1.068,1.133)
1. Dropped Activities and Interests						
No	776,219	22,454 (2.89)	4,025,237.37	5.5783	1 (ref.)	1 (ref.)
Yes	146,550	4,832 (3.30)	781,025.24	6.18674	1.097 (1.063,1.132)	1.090 (1.057,1.125)
2. Worthlessness						
No	856,074	24,996 (2.92)	4,448,360.15	5.61915	1 (ref.)	1 (ref.)
Yes	66,695	2,290 (3.43)	357,902.47	6.39839	1.125 (1.078,1.175)	1.115 (1.068,1.164)
3. Hopelessness						
No	850,549	24,783 (2.91)	4,420,542.38	5.60633	1 (ref.)	1 (ref.)
Yes	72,220	2,503 (3.47)	385,720.24	6.48916	1.145 (1.099,1.193)	1.135 (1.089,1.183)
Clinically diagnosed depressive disorder						
No	892,956	26,206 (2.93)	4,654,299.5	5.63049	1 (ref.)	1 (ref.)
Yes	29,813	1,080 (3.62)	151,963.11	7.10699	1.267 (1.192,1.346)	1.241 (1.167,1.319)
No recur	20,922	713 (3.41)	107,120.88	6.65603	1.184 (1.098,1.275)	1.164 (1.080,1.254)
Recur within 2 years	8,891	367 (4.13)	44,842.23	8.18425	1.468 (1.325,1.628)	1.421 (1.281,1.576)

Model 1: adjusted for age and gender.

Model 2: adjusted for age, gender, smoking, drinking, exercise, income, body mass index, hypertension, diabetes, and hyperlipidemia.

IR: incidence rate.

Subjects with at least one subjective depressive symptom had increased hazard of developing glaucoma after adjusting for confounders (adjusted HR = 1.10 [95% CI, 1.07–1.13]) than those without depressive symptoms. Subjects with clinically diagnosed depressive disorder within 1 year prior to NSPTA showed even higher risk of glaucoma (adjusted HR = 1.24 [95% CI, 1.17–1.32]) than those without depressive disorder. In subgroup analysis, glaucoma risk was higher in those whose depressive disorder recurred (adjusted HR = 1.42 [95% CI, 1.28–1.58]) than those who did not (adjusted HR = 1.16 [95% CI, 1.08–1.25]).

Further glaucoma risk stratification was attained according to the depression status: subjective depressive symptoms at the time of NSPTA, clinically diagnosed depressive disorder within one year prior to NSPTA, or both (Table 3). The risk of glaucoma increased sequentially from those with no depression-neither subjective depressive symptom nor clinically diagnosed depressive disorder- to those with subjective depressive symptom (adjusted HR = 1.09 [95% CI, 1.06–1.13]), those with clinically diagnosed depressive disorder (adjusted HR = 1.23 [95% CI, 1.14–1.32]), and those with both subjective depressive symptom and clinically diagnosed depressive disorder (adjusted HR = 1.36 [95% CI, 1.22–1.52]). In addition, those whose clinically diagnosed depressive disorder recurred showed higher risk of glaucoma than those whose disorder did not.

**Table 3:** Combined risk of glaucoma according to subjective depressive symptom and clinically diagnosed depressive disorder.

Group	Subjective depressive symptom*	Clinically diagnosed depressive disorder	N	Glaucoma N (%)	Duration (person years)	IR per 1000	HR (95% C.I)	
							Model 1	Model 2
No depression	No	No	731,133	20,910 (2.86)	3,791,759.22	5.51459	1 (ref.)	1 (ref.)
Depression	Yes	No	161,823	5,296 (3.27)	862,540.28	6.14	1.101 (1.068,1.135)	1.094 (1.062,1.128)
	No	Yes	21,580	749 (3.47)	109,279.6	6.85398	1.247 (1.159,1.342)	1.225 (1.139,1.319)
		No recur	15,451	510 (3.30)	78,575.44	6.49058	1.179 (1.079,1.288)	1.162 (1.064,1.27)
		Recur	6,129	239 (3.90)	30,704.16	7.78396	1.421 (1.251,1.615)	1.383 (1.217,1.572)
	Yes	Yes	8,233	331 (4.02)	42,683.51	7.75475	1.405 (1.26,1.567)	1.361 (1.220,1.518)
		No recur	5,471	203 (3.71)	28,545.44	7.11147	1.283 (1.117,1.474)	1.247 (1.085,1.433)
		Recur	2,762	128 (4.63)	14,138.07	9.05357	1.648 (1.385,1.961)	1.585 (1.331,1.888)

Model 1: adjusted for age and gender.

Model 2: adjusted for age, gender, smoking, drinking, exercise, income, body mass index, hypertension, diabetes, and hyperlipidemia.

IR: incidence rate.

* At least one of the three depressive symptoms from the Geriatric Depression Scale.

## Discussion

The purpose of this study was to evaluate the risk of incident glaucoma in patients with depression using a longitudinal nationwide database. To the best of our knowledge, this is the first study to evaluate the temporal association between depression and glaucoma development.

The results of our study revealed that subjects with depression have a higher prospective risk of developing glaucoma compared to those without depression, and this relationship remained after adjusting for potential confounding factors including age, gender, smoking, drinking, exercise, income, body mass index, hypertension, diabetes, and dyslipidemia.

Previous studies have shown that depression increased the risk of other chronic diseases including coronary artery disease^2,3^, hypertension^2,5–7^, diabetes^8–10^, asthma^2^, migraine^2,11^, chronic bronchitis^2^, and back pain^38^. Depressive symptoms were reported to increase the risk of hypertension in young adults in the CARDIA (Coronary artery development in young adults) study^5^. The authors suggested that alterations in adrenergic activity in clinical depression may increase blood pressure. Breslau et al.^11^ reported that major depression increased the risk of migraine, but not other headaches, in a longitudinal cohort study and proposed that the two diseases share etiology relating to hormonal or neurotransmitter systems. Patten et al.^2^ reported that depression increased the risk of several medical conditions, but not cataract and glaucoma. However, in their study, they categorized cataract and glaucoma into one disease entity, so relationship between depression and glaucoma is not investigated thoroughly in their study.

Although the underlying mechanism between depression and development of glaucoma is unclear, both share several common pathophysiology which may explain the association. In glaucoma, intraocular pressure causes mechanical strain on the optic nerve head, which results in disruption of axonal transport. Decreased retrograde transport of essential trophic factors including BDNF contribute to RGC loss^25,26^. In depression, neuroplasticity, which is mediated by regulatory proteins, such as BDNF, is disrupted^23,24^. Diminished level of BDNF in depression may affect the survival of RGC in glaucoma development.

Neuroinflammation also plays a role in the pathophysiology of both diseases^24^. Neuroinflammation and microglial activation have been found in the brains of patients with depression^29^, and inflammatory cytokines such as c-reactive protein, interleukin 6, and tumor necrosis factor (TNF) have been reported to be increased^39^. Also in glaucoma, there is mounting evidence that neuroinflammation plays a role in its pathogenesis^30–32^. Microglial activation has been reported in glaucomatous optic nerve heads^40–42^, and TNF alpha, interleukins, and various inflammatory cytokines are also involved in glaucoma^43–45^.

In addition, autonomic nervous system function is often altered in depression, and this seems to be the link between depression and other physical dysfunctions^33–35^. Heart rate variability (HRV), which is one of the most widely used methods to measure autonomic dysfunction, is shown to be lower in patients with depression compared with controls^46^. Autonomic dysfunction as shown by low HRV has also been associated with glaucoma, especially those with normal tension, which is the most prevalent type of glaucoma in Korea^36,37^. This may also partly account for the increased risk of glaucoma in depression patients.

Furthermore, unhealthy lifestyle behaviors related with depression may have influenced our results. Depression is associated with unhealthy lifestyle behaviors including smoking, drinking, and physical inactivity^47–49^. Of note, patients with depression have been found to have increased risk of being obese, which may be attributed to genetics, alterations in homeostatic adjustments such as hypothalamic–pituitary–adrenal axis or immune responses, and antidepressants^50^. These lifestyle behaviors and obesity have been associated with elevated intraocular pressure or increased risk of developing glaucoma^51,52^. However, in our study, depression increased the risk of glaucoma regardless of smoking, drinking, exercise status, or BMI.

The strengths of this study include its nationwide database, all subjects aged 66, which minimize selection bias thus resulting in higher generalizability. In addition, our study used ICD-10 diagnostic codes to identify individuals with depression and glaucoma, which minimizes overestimation of both diseases. Furthermore, we excluded those who were diagnosed with glaucoma one year prior to NSPTA and investigated the risk of glaucoma onset in individuals with depression, thereby establishing temporal relationship between depression and glaucoma.

However, there are also some limitations. First, our study is hampered by its retrospective design. Also, there is potential for surveillance bias in the diagnosis of glaucoma indicating that individuals with depression may have higher rate of consultation with health professionals, increasing the chance of glaucoma diagnosis. Second, we were unable to determine whether antidepressants contributed to glaucoma incidence. Third, despite the statistical significance, the Cohen *d* were all less than 0.2 indicating small effect sizes. However, this large sample enabled us to find the relationship between depression and glaucoma. Fourth, although we were able to reveal temporal relationship and also graded relationship (*i.e.* those with more severe depression represented by diagnosed depressive disorder and recurred diagnosis showed increased risk of glaucoma) between depression and incident glaucoma, this can hardly provide a logical conclusion on causality between depression and glaucoma onset. Last, we did not compare our results to or control for other psychiatric diagnoses. These limitations warrant further investigation.

In conclusion, in this nationwide population-based cohort study, we found that individuals with depression had a greater risk of developing glaucoma than those without depression. Subjective depressive symptoms and clinically diagnosed depressive disorder independently and synergistically increased the risk of glaucoma incidence. Further research into the mechanisms through which these illnesses are associated is warranted.

## Methods

### Data source

In this nationwide population-based cohort study, the KNHID was provided by the National Health Insurance Service (KNHIS, NHIS-2019-1-556). The KNHIS requires all nationals to enroll in the system and KNHID includes comprehensive health-related information about demographic characteristics (anonymized code for each individual, age, gender, socioeconomic variables, household income level, etc.), and medical data based on medical claims (diagnostic codes by the International Classification of Diseases 10th revision [ICD-10], admission and ambulatory care, treatment procedures, and prescription records). KNHIS also conducts National Health Screening Program (NHSP), which includes a questionnaire (health-related lifestyle and medical history), basic physical data (body mass index [BMI], waist circumference, systolic/diastolic blood pressure, etc.), and laboratory examination (fasting glucose, triglyceride, etc.) to all workplace subscribers and their dependents and to all Koreans over the age of 40 at least biennially^53^.

Furthermore, additional health screening is conducted for the ages of 40, representing transition to middle age, and 66, representing transition to elderly, which is called the National Screening Program for Transitional Ages (NSPTA). NSPTA includes questionnaires regarding depressive symptoms, which are 3 selected questions from the geriatric depression scale (GDS), in addition to NHSP.

### Study design

In this study, we included subjects who participated in the NSPTA during 2009–2014 at the age of 66. These subjects were divided into depression group and no depression group and were tracked until 2017 for development of glaucoma. Those who were diagnosed with glaucoma within one year prior to NSPTA were excluded from the analysis.

Depression was defined as having either clinically diagnosed depressive disorder or subjective depressive symptoms. Depressive disorder was defined using claims data based on ICD-10 code for major depressive disorder (F32x or F33x) within one year prior to NSPTA. Those with depressive disorders were further categorized into those with no recur during the follow-up and those whose depressive disorder recurred within 12–24 months after the first diagnosis. To evaluate subjective depressive symptoms, three questions from the GDS were used: “Have you dropped many of your activities and interests?” “Do you feel pretty worthless the way you are now?” “Do you feel that your situation is hopeless?” representing one’s activities/interest, worthlessness, and hopelessness, respectively. Subjects who answered “No” to all three questions were defined as no subjective depressive symptom, and those with at least one “Yes” were defined as having subjective depressive symptom.

Glaucoma was defined based on ICD-10 code for primary open-angle glaucoma (H401), and to enhance the validity of the diagnosis, those with at least 3 records with glaucoma diagnosis were included in the study^54^. This study was approved by the Institutional Review Board of the Yeouido St. Mary’s Hospital, Seoul, Korea, which waived consent from individual subjects because we used publicly open and anonymized data. Our research adhered to the tenets of the Declaration of Helsinki.

### Statistical analysis

To compare continuous and categorical variables, student’s t-test and χ^2^ test were used, respectively. The follow-up duration (person-years) was calculated from the date of NSPTA until the date of glaucoma diagnosis or until the end of follow-up in December 2017. Cox proportional hazards analysis was performed to calculate the hazard ratio (HR) for the association between depression and glaucoma incidence before and after adjusting for potential confounding factors. Fully-adjusted model included age, gender, smoking, alcohol, physical exercise, income, body mass index, diabetes, hypertension, and dyslipidemia as potential confounding factors. In addition, to relate the influence of depression to other lifestyle factors, we also calculated the HR for glaucoma development based on combination of depression and other lifestyle factors including smoking and alcohol. The effect size was calculated using Cohen *d* following the equation: d = ln (HR) × √ 6 / π^55^.

We used SAS ver 9.4 (SAS Institute, Cary, NC, USA) for all statistical analysis with *P-*values < 0.05 considered significant.

## Supplementary Information

Supplementary Information

## Data Availability

Data are available from the Korea National Health Insurance Sharing Service Institutional Data Access Committee (https://nhiss.nhis.or.kr/bd/ay/bdaya001iv.do) for researchers who meet the access criteria.
