# Supplementary material for: Effect of depressive symptom and depressive disorder on glaucoma incidence in elderly
Source: Sci Rep. 2021 Mar 15;11:5888. doi: 10.1038/s41598-021-85380-6 (PMC7961135; doi:10.1038/s41598-021-85380-6)
Supplement: Supplementary file 1 — Supplementary Information [file 41598_2021_85380_MOESM1_ESM.docx]

Effect of depressive symptom and depressive disorder on glaucoma incidence in elderly

Younhea Jung, MD, PhD^a^, Kyungdo Han, PhD^b^, Sheng-min Wang, MD, PhD^c^, Hye yeon Yoon, MD^a^, Jung Il Moon, MD, PhD^a^*

^a^Department of Ophthalmology, Yeouido St. Mary’s Hospital, College of Medicine, The Catholic University of Korea, Seoul, Korea

^b^Department of Statistics and Actuarial Science, Soongsil University, Seoul, Republic of Korea

^c^Department of Psychiatry, Yeouido St. Mary’s Hospital, College of Medicine, The Catholic University of Korea, Seoul, Korea

**Correspondence:** Jung Il Moon, MD, PhD

Department of Ophthalmology, Yeouido St. Mary’s Hospital, College of Medicine, The Catholic University of Korea, 10, 63-ro, Yeongdeungpo-gu, Seoul, 07345, Republic of Korea

Tel: 82-2-3779-1243, E-mail: [jimoon@catholic.ac.kr](mailto:jimoon@catholic.ac.kr)

Supplementary Table 1. Number of subjects with glaucoma

|  | Glaucoma  N(%) |
| --- | --- |
| N = 922,769 | 27,286(2.96) |
| Age |  |
| Gender, Male | 13,036(2.86) |
| Income (low) | 8,105(3.05) |
| Diabetes (yes) | 6,723(3.73) |
| Hypertension (yes) | 15,474(3.15) |
| Hyperlipidemia (yes) | 10,272(3.19) |
| Smoking |  |
| Never | 18,706(3) |
| Ex-smoker | 5,264(3.12) |
| Current smoker | 3,276(2.53) |
| Alcohol |  |
| Non | 19,130(2.99) |
| Mild to Moderate | 6,881(2.88) |
| Heavy | 1,113(2.74) |
| Exercise |  |
| Heavy (≥ 3 days) | 5,619(3.09) |
| Moderate (≥ 5 days) | 7,148(3.06) |
| Light (≥ 5 days) | 14,761(3.02) |

|  | Depression | N | Glaucoma | Duration (person years) | IR per 1000 | HR(95% CI) | | |
| --- | --- | --- | --- | --- | --- | --- | --- | --- |
|  |  |  |  |  |  | Model 1 | Model 2 | Model 2 |
| Smoking |  |  |  |  |  |  |  |  |
| Never | No | 491,087 | 14,265 | 2,573,667.85 | 5.54267 | 1(Ref.) | 1(Ref.) | 1(Ref.) |
|  | Yes | 132,220 | 4,441 | 709,231.73 | 6.26171 | 1.117(1.08,1.155) | 1.106(1.069,1.144) | 1.107(1.07,1.145) |
| Ex-smoker | No | 137,584 | 4,149 | 692,347.2 | 5.99266 | 1.116(1.072,1.163) | 1.101(1.057,1.148) | 1(Ref.) |
|  | Yes | 31,294 | 1,115 | 159,778.78 | 6.9784 | 1.287(1.207,1.373) | 1.257(1.178,1.341) | 1.147(1.072,1.226) |
| Current smoker | No | 101,661 | 2,471 | 520,366.2 | 4.74858 | 0.875(0.835,0.918) | 0.866(0.826,0.909) | 1(Ref.) |
|  | Yes | 27,725 | 805 | 142,813.77 | 5.63671 | 1.037(0.963,1.116) | 1.014(0.941,1.092) | 1.151(1.062,1.248) |
|  |  |  |  |  |  |  |  | P for interaction 0.345 |
| Alcohol drinking |  |  |  |  |  |  |  |  |
| Non | No | 499,637 | 14,445 | 2,600,236.4 | 5.55526 | 1(Ref.) | 1(Ref.) | 1(Ref.) |
|  | Yes | 139,437 | 4,685 | 740,579.93 | 6.32612 | 1.13(1.093,1.168) | 1.117(1.08,1.154) | 1.114(1.077,1.151) |
| Mild to Moderate | No | 195,470 | 5,489 | 1,000,450.59 | 5.48653 | 0.996(0.962,1.03) | 0.989(0.955,1.024) | 1(Ref.) |
|  | Yes | 43,043 | 1,392 | 224,607.84 | 6.19747 | 1.113(1.052,1.177) | 1.105(1.044,1.17) | 1.124(1.059,1.193) |
| Heavy | No | 32,498 | 855 | 167,734.12 | 5.09735 | 0.922(0.859,0.99) | 0.923(0.859,0.992) | 1(Ref.) |
|  | Yes | 8,054 | 258 | 41,965.17 | 6.14796 | 1.103(0.974,1.25) | 1.102(0.972,1.249) | 1.209(1.051,1.392) |
|  |  |  |  |  |  |  |  | P for interaction 0.655 |

Supplementary Table 2. Effect of depression and lifestyle factors on glaucoma incidence

Model 1: adjusted for age and gender

Model 2: adjusted for age, gender, smoking, drinking, exercise, income, body mass index, hypertension, diabetes, and hyperlipidemia

IR: incidence rate

HR: Hazard ratio

CI: Confidence interval
